# Differentiation markers in pancreatic head adenocarcinomas: MUC1 and MUC4 expression indicates poor prognosis in pancreatobiliary differentiated tumours

**DOI:** 10.1111/j.1365-2559.2009.03227.x

**Published:** 2009-02

**Authors:** Arne Westgaard, Aasa R Schjølberg, Milada Cvancarova, Tor J Eide, Ole Petter F Clausen, Ivar P Gladhaug

**Affiliations:** 1 Faculty of Medicine, University of Oslo, Rikshospitalet University HospitalOslo; 2 Division of Surgery University HospitalOslo, Norway; 3 Division of Pathology University HospitalOslo, Norway; 4 Biostatistics Unit, Department of Research Services, Rikshospitalet University HospitalOslo, Norway

**Keywords:** histological differentiation, immunohistochemical markers, intestinal histological type, pancreatic head adenocarcinoma, pancreatobiliary histological type

## Abstract

**Aims:**

To examine how accurately immunohistochemical markers discriminate between pancreatobiliary and intestinal-type adenocarcinomas in the pancreatic head and to explore the prognostic importance of these markers among each of these histological types.

**Methods and results:**

Histopathological features of 114 consecutively resected adenocarcinomas of pancreatobiliary (*n* = 67) and intestinal (*n* = 47) type of differentiation were recorded according to a standardized protocol. Immunohistochemistry for cytokeratin (CK) 7, CK20, MUC1, MUC2, MUC4 and CDX2 was performed on tissue microarrays. Classification of the adenocarcinomas based on immunohistochemistry was compared with the morphological evaluation of histological type. Presence of CK7 and MUC4, and absence of CDX2, were independent predictors of pancreatobiliary versus intestinal type. Using these markers to optimize immunohistochemical classification, agreement between immunohistochemical and morphological classification was only moderate (κ = 0.53). In pancreatobiliary differentiated tumours, MUC1 and/or MUC4 expression was an independent prognostic factor (hazard ratio 2.02, 95% confidence interval 1.02, 3.98) when adjusting for nodal involvement, vessel involvement and tumour size. In intestinally differentiated tumours, none of the markers was significantly associated with prognosis.

**Conclusions:**

Agreement between immunohistochemical and morphological classification of pancreatic head adenocarcinomas is moderate. In pancreatobiliary adenocarcinomas, MUC1 and/or MUC4 expression indicates a particularly poor prognosis.

## Introduction

Resectable adenocarcinomas in the pancreatic head may originate from pancreatic, ampullary, distal bile duct or duodenal tissue.^1^,^2^ The histological appearance of these adenocarcinomas, typically either pancreatobiliary or intestinal, often resembles the site of origin.^3^ However, ampullary,^4^,^5^ distal bile duct^6^,^7^ and pancreatic tumours^8^,^9^ may have features of both phenotypes, and duodenal adenocarcinomas may be impossible to discriminate from intestinal type ampullary adenocarcinomas.^4^ In a previous report,^10^ we found that the histological type of differentiation, pancreatobiliary or intestinal, was a better independent predictor of survival than the anatomical site of tumour origin, which is often difficult to determine.^4^,^11^–^16^

In addition to standard histopathological examination of the resected pancreatoduodenectomy specimens, immunohistochemical examination with antibodies directed against differentiation markers may be applied to discriminate between tumours of pancreatobiliary and intestinal-type differentiation.^6^,^8^,^9^,^17^–^26^ No single marker has so far been found to distinguish reliably between the two histological types and several differentiation markers are often used in combination.^6^,^22^–^26^ The differentiation markers most often used are cytokeratin (CK) 7 and CK20, and the mucin proteins MUC1 and MUC2. More recently, expression of the CDX2 homeodomain protein, important for intestinal differentiation in normal gastrointestinal tissue,^20^,^27^ has been shown to be associated with intestinal-type differentiation and with improved prognosis in pancreatic head adenocarcinomas.^6^,^8^,^9^,^19^,^22^,^25^,^26^,^28^,^29^ The mucin protein MUC4 has been found to be aberrantly expressed in pancreatic adenocarcinoma^21^,^30^–^32^ and in extrahepatic bile duct carcinoma,^33^ but less frequently expressed in the normal epithelium of the duodenum and ampulla.^34^,^35^

The aim of the present study was to compare immunohistochemical markers with the morphological classification of the histological type of differentiation and to evaluate their prognostic importance in a cohort of resected pancreatic head adenocarcinomas from all four anatomical origins.

## Methods

### Patients

Permission for the study was obtained from the National Committee for Research Ethics. The study comprised all patients (*n* = 114) with primary pancreatic head adenocarcinoma of pancreatobiliary (*n* = 67) or intestinal (*n* = 47) histological type who underwent a pancreaticoduodenectomy with curative intent (R0 and R1 resections) during 1998–2004 at Rikshospitalet University Hospital. Most tumours originated in the pancreas (*n* = 40) or the ampulla of Vater (*n* = 41). Less than one-third originated in the distal bile duct (*n* = 17) or the duodenum (*n* = 16). Perioperative death (in-hospital death or death within 30 days of operation; *n* = 4) was included in the association analysis, but excluded from survival analysis. Analysis including perioperative death gave very similar results. No patients were lost to follow-up. Details of the patient cohort have been previously published.^11^

### Histopathological assessment of specimens

Histological type of differentiation, tumour origin, pT stage, maximum tumour diameter, resection status, nodal involvement, perineural infiltration, vascular involvement and degree of differentiation were prospectively registered according to a standardized protocol for histopathological examination, and retrospectively re-evaluated by an experienced pathologist, as previously described.^11^ The histological type of differentiation was classified according to criteria first suggested by Kimura *et al.*,^5^ later revised by Albores-Saavedra *et al.*^4^ Cases with mixed type differentiation were classified according to the dominant pattern.^5^,^10^ All tumours were assigned to one of these two histological types of differentiation. Details of the classification have been previously published.^10^

### Immunohistochemistry

Paraffin-embedded tissue specimens were used for construction of tissue microarrays, using a Manual Tissue Arrayer MTA-1 (Beecher Instruments, Inc., Sun Prairie, WI, USA). The arrays contained two to four 1.0-mm cores from each tumour. Blocks were serially sectioned at 4 μm thickness, put on slides and stored. The sections were deparaffinized and pretreated before incubation with mouse monoclonal antibodies directed against CK7, CK20, MUC1, MUC2, MUC4 and CDX2 (Table 1). The mucins MUC1, MUC2 and MUC4 are heavily glycosylated and changes in tissue glycosylation can modify the reactivity to the antibodies without changes in the *MUC* gene product at the protein level. The specificity of the antibodies used in the present study to detect MUC1 (clone Ma695^36^) and MUC2 (clone Ccp58^37^) was, according to the manufacturer: MUC1, carbohydrate epitope of the human MUC1 glycoprotein; MUC2, human MUC2 glycoprotein; however, no epitope mapping studies have been published for these antibodies. The MUC4 antibody used in the present study (clone 1G8^38^) has been shown to react with human MUC4β and recognizes an epitope on the polypeptide chain rather than a carbohydrate epitope.^38^ All immunohistochemistry was performed with a Ventana Nexus Autostainer using a Ventana iVIEW Diaminobenzidene Detection kit, according to the manufacturer’s protocol (Ventana Medical Systems Inc., Tucson, AZ, USA). The sections were counterstained using Harris's haematoxylin for 10 s. Positive and negative control tissues were included in the tissue microarray blocks.

**Table 1:** Primary antibodies used for immunohistochemistry

Antigen	Antibody clone	Manufacturer	Dilution	HIER
CK7	OV-TL 12/30	Dako*	1:50	Tris–EDTA

CK20	Ks20.8	Dako*	1:50	Tris–EDTA

MUC1	NCL-Muc-1, Ma695	Novocastra†	1:50	Citrate

MUC2	NCL-Muc-2, Ccp58	Novocastra†	1:50	Citrate

MUC4	1G8	Zymed‡	1:50	Citrate

CDX2	CDX-88	BioGenex§	1:50	Tris–EDTA

Pretreatment was performed by exposing the slides to 0.5% H_2_O_2_ for 10 min, followed by antigen retrieval with Tris–EDTA pH 9 or citrate pH 6 in a microwave oven: heat to boiling, then reduced to ‘keep warm’ for 30 min.

HIER, Heat-induced epitope retrieval; CK, Cytokeratin; EDTA, Ethylenediamine tetraaceticacid.

* DakoCytomation Denmark A/S, Glostrup, Denmark.

† Novocastra Laboratories Ltd., Newcastle upon Tyne, UK.

‡ Zymed Laboratories, Invitrogen Immunodetection, South San Francisco, CA, USA.

§Biogenex Laboratories Inc., San Ramon, CA, USA.

Representative tissue was obtained for all except one tumour for CK7, MUC4 and CDX2, and for all except three tumours for CK20 and MUC1. For CDX2, only nuclear immunoreactivity was considered and the percentage of positive nuclei was registered on a continuous scale. For the remaining markers, cytoplasmic or membranous reactivity was classified as 0 (no reactivity), 1 (reactivity in <10% of tumour cells), 2 (reactivity in >10% but <40% of tumour cells), and 3 (reactivity in >40% of tumour cells). Intensity of immunoreactivity was also registered, but not used for scoring.

The level of expression representing a positive sample was defined prior to statistical analysis. Tumours were classified as CDX2+ if nuclear reactivity was seen in any tumour cell in the sample. For all the other markers, tumours were classified as positive if there was cytoplasmic reactivity of ≥10% of the tumour cells. Histograms (for nuclear CDX2 reactivity) and bar graphs (for the semiquantitatively scored markers), comparing the level of marker expression with the histological type for each tumour, were examined to verify that the predefined cut-off values were relevant (data not shown). For the survival analysis, the markers were additionally evaluated using each of the semiquantitative scores (categories 0–3) as cut-off points or as a continuous variable (for CDX2).

### Statistical analysis

Associations between each immunohistochemical marker and the histological type were evaluated using Fisher’s exact test. Interobserver agreement was estimated using Cohen’s κ and categorized as poor (κ < 0.20), fair (0.21 < κ < 0.40), moderate (0.41 < κ < 0.60), substantial (0.61 < κ < 0.80), or almost perfect (κ > 0.80). Binary logistic regression with forward variable selection was performed to identify independent markers of the histological type of differentiation. Bar graphs showing the combined expression of each of these independent markers versus the histological type were examined to identify the phenotypes that optimized agreement between immunohistochemical and morphological classification. Survival data were obtained from the National Registry of Norway, updated 28 January 2008. Only overall survival was considered. Follow-up was limited to 5 years. By the end of the study, 79 of 114 patients were dead; only nine patients were followed for <5 years (range 3.1–4.8 years). The Kaplan–Meier method, the log rank test and Cox regression analysis were used to assess overall survival. For all tests, a two-sided *P* < 0.05 was considered to be statistically significant. Statistical analyses were performed using SPSS 15.0 for Windows software (SPSS Inc., Chicago, IL, USA).

## Results

### Classification of pancreatic head adenocarcinomas based on immunohistochemistry and morphology

**Figure 1:**
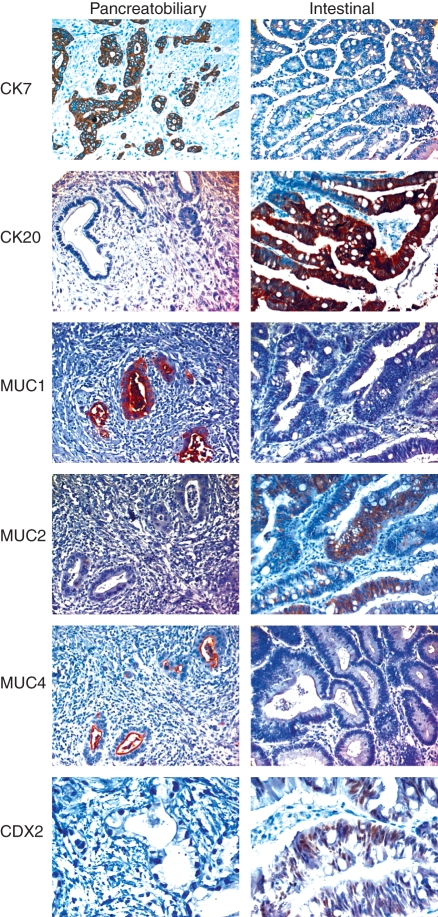
Typical staining for differentiation markers in pancreatic head adenocarcinomas with pancreatobiliary [left column; positive for cytokeratin (CK) 7, MUC1 and MUC4] and intestinal (right column; positive for CK20, MUC2 and CDX2) type of histological differentiation. CK7 and CK20 immunoreactivity is intense and localized to the cytoplasm. Reactivity for MUC1 is less intense and localized both to the cytoplasm and/or the cell membrane, whereas MUC2 reactivity is localized to the cytoplasm. MUC4 reactivity is predominantly membranous, although some cytoplasmic reactivity is also seen. Only nuclear CDX2 reactivity is associated with the histological type of differentiation. In intestinal adenocarcinomas, areas with transition from negative to positive reactivity in a single tumour gland can often be seen, as demonstrated here for CK20 and MUC2.

**Figure 2:**
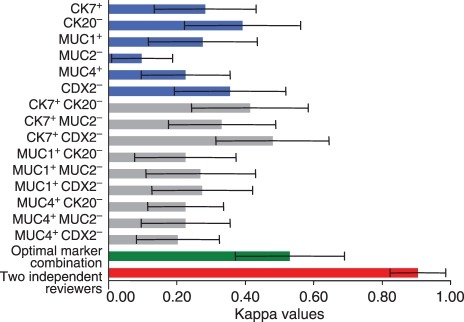
Comparison of histological and immunohistochemical classification of pancreatic head adenocarcinomas using κ statistics (with 95% confidence intervals). Red bar represents interobserver agreement between two independent reviewers of the histological type (reported previously^10^). The other bars compare histological classification (pancreatobiliary versus intestinal) with immunohistochemical classification (IHC pancreatobiliary versus IHC intestinal; single markers, blue bars; marker combinations, grey bars; the optimized marker combination, green bar).

**Figure 3:**
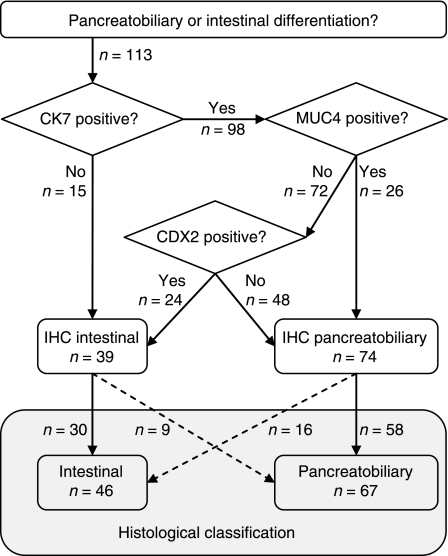
Algorithm for classification of pancreatobiliary versus intestinal type using immunohistochemical markers independently associated with histological type. Optimized immunohistochemical classification (IHC) corresponds to morphological classification in 88 of 113 tumours (78%); i.e. 58 pancreatobiliary tumours and 30 intestinal tumours were correctly identified by immunohistochemical evaluation [positive predictive value for identification of pancreatobiliary versus intestinal histological type was thus 78%, 95% confidence interval (CI) 67, 87; sensitivity was 87%, 95% CI 76, 93, and specificity was 65%, 95% CI 50, 78].

Figure 1 shows a typical pattern of immunoreactivity for the differentiation markers in pancreatic head adenocarcinomas. As detailed in Table 2, CK7, MUC1 and MUC4 reactivity was more often positive in pancreatobiliary-type adenocarcinomas, whereas MUC2, CK20 and CDX2 reactivity was more often positive in intestinal-type adenocarcinomas (*P* < 0.05 for each marker). None of these molecules identified pancreatobiliary or intestinal-type tumours with sufficient sensitivity and specificity to yield high predictive values (Table 2). Interobserver agreement between two independent reviewers evaluating the histological type by morphological criteria was almost perfect [κ = 0.90; 95% confidence interval (CI) 0.82, 0.99; Figure 2, red bar], as shown previously.^10^ Comparing immunohistochemical and morphological classification of the histological type (pancreatobiliary versus intestinal), agreement was only fair to moderate (κ < 0.40 for each marker; upper 95% CI < 0.60; Figure 2, blue bars). Evaluation using alternative cut-off values did not considerably improve agreement (data not shown). Some improvement was seen when evaluating the combined expression of two markers (Figure 2, grey bars), particularly using the phenotype CK7+ CDX2− to identify pancreatobiliary adenocarcinomas (κ = 0.48; 95% CI 0.31, 0.65). Binary logistic regression revealed that CK7+ (*P* = 0.009), CDX2− (*P* = 0.002) and MUC4+ (*P* = 0.026) were independent markers of the pancreatobiliary type. Bar graphs showing these three markers versus morphological histological type were therefore examined to obtain the optimal combination of differentiation markers (Figure S1) to classify tumours as either immunohistochemically pancreatobiliary (‘IHC pancreatobiliary’) or immunohistochemically intestinal (‘IHC intestinal’). The phenotypes CK7+ MUC4+ and CK7+ CDX2− optimally classified tumours as IHC pancreatobiliary, and the phenotypes CK7− and MUC4− CDX2+ optimally classified tumours as IHC intestinal (Figure 3). The positive predictive value for identification of pancreatobiliary versus intestinal histological type was 78% (95% CI 67, 87); sensitivity 87% (95% CI 76, 93), specificity 65% (95% CI 50, 78). However, even with these optimized combinations, the κ-value for immunohistochemical versus morphological classification was only moderate (0.53; 95% CI 0.37, 0.69; Figure 2, green bar).

**Table 2:** Tumour markers in detection of pancreatobiliary versus intestinal histological type of differentiation in pancreatic head adenocarcinomas

	Histological type								
								
	Pancreatobiliary		Intestinal						
							
	Pos	Neg	Pos	Neg	Sensitivity, %	Specificity, %	*P*-value*	PPV (%)	95% CI
Pancreatobiliary markers									
CK7	65	2	33	13	97	28	<0.001	66	[56–75%]

MUC1	32	33	9	37	49	80	0.001	78	[62–89%]

MUC4	23	44	4	42	34	91	0.002	85	[65–95%]

Intestinal markers									
CK20	17	48	31	15	67	74	<0.001	65	[49–77%]

MUC2	1	64	6	40	13	98	0.020	86	[42–99%]

CDX2	10	57	25	21	54	85	<0.001	71	[53–85%]

PPV, Positive predictive value (i.e. the probability of obtaining a true positive test result); CI, Confidence interval; Pos, Immunopositivity; Neg, Immunonegativity (any nuclear reactivity was considered a positive sample for CDX2, whereas cytoplasmic reactivity in >10% of tumour cells defined a positive sample for the remaining markers); CK, Cytokeratin.

* Fisher’s exact test, *P*-value for each marker versus the histological type determined morphologically.

### Survival analysis

**Figure 4:**
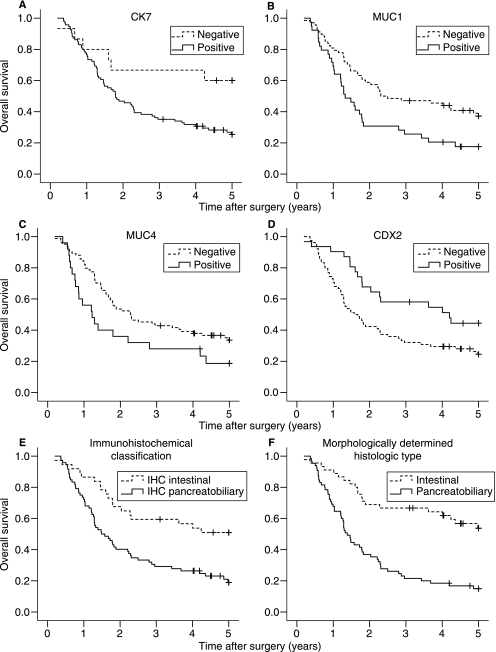
Overall survival after pancreaticoduodenectomy predicted by markers associated with histological type and by histology alone. **A**, Cytokeratin (CK) 7+ (*n* = 94) versus CK7− (*n* = 15); *P* = 0.029. **B**, MUC1+ (*n* = 39) versus MUC1− (*n* = 68); *P* = 0.008. **C**, MUC4+ (*n* = 25) versus MUC4− (*n* = 84); *P* = 0.041. **D**, CDX2− (*n* = 78) versus CDX2+ (*n* = 31); *P* = 0.019. **E**, IHC pancreatobiliary type (CK7+ MUC4+ or CK7+ CDX2−, *n* = 72) versus IHC intestinal type (CK7− or MUC4− CDX2+, *n* = 37); *P* = 0.001. **F**, Histological type (updated from^10^), pancreatobiliary (*n* = 65) versus intestinal (*n* = 45); *P* < 0.001.

On univariate analysis of all pancreatic head adenocarcinomas (Figure 4), expression of the pancreatobiliary type markers CK7, MUC1 and MUC4 predicted a poor prognosis, whereas expression of the intestinal marker CDX2 was significantly associated with better survival (Figure 4A–D, *P* < 0.05 for each marker). Combining markers for the optimal immunohistochemical classification of the histological type, IHC pancreatobiliary type significantly predicted poor survival compared with IHC intestinal type (Figure 4E, *P* = 0.001). Survival for pancreatobiliary versus intestinal type based on morphological evaluation is shown in Figure 4F (*P* < 0.001).

**Figure 5:**
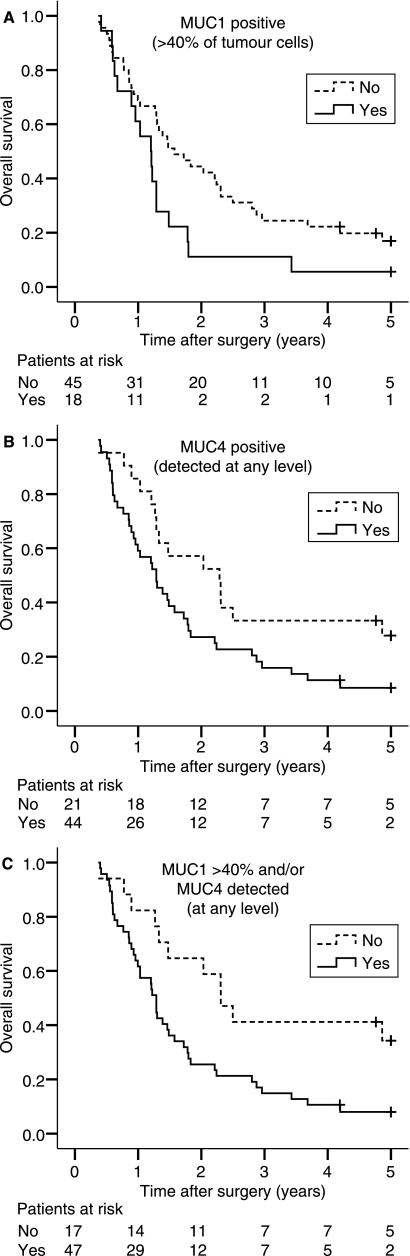
Overall survival after pancreaticoduodenectomy for pancreatobiliary differentiated adenocarcinomas. **A**, MUC1 expression in >40% of tumour cells (*n* = 18) versus <40% or no MUC1 expression (*n* = 45); *P* = 0.038. **B**, Any MUC4 expression (*n* = 44) versus no MUC4 expression (*n* = 21); *P* = 0.029. **C**, MUC1 >40% and/or MUC4 >0%, yes (*n* = 47) versus no (*n* = 17); *P* = 0.009.

In morphologically determined pancreatobiliary-type adenocarcinomas (*n* = 67), most tumours (58 of 65) had diffuse CK7 expression (in >40% of the tumour cells) or were largely negative for MUC2 (62 of 65) and CDX2 (57 of 65). These markers were not analysed further. CK20, MUC1 and MUC4 were variably expressed in pancreatobiliary adenocarcinomas (Table 3). Whereas CK20 was non-significant, MUC1 expression in ≥40% of tumour cells (Figure 5A, *P* = 0.038) and MUC4 expression in any tumour cell (Figure 5B, *P* = 0.029) each significantly predicted a poor prognosis. Combining MUC1 and/or MUC4 expression at these levels (MUC1 > 40% and/or MUC4 > 0%, versus MUC1 < 40% and MUC4 = 0%) indicated a clear survival disadvantage for patients with pancreatobiliary differentiated adenocarcinomas (Figure 5C, *P* = 0.009). Expression of one or both of these markers at these levels was significantly associated with pancreatic versus non-pancreatic tumour origin (*P* = 0.001), while not associated with lymph node or resection margin involvement, vascular or perineural infiltration, degree of differentiation, tumour size or pT stage (data not shown). Starting with all these factors in a multivariable Cox regression analysis, with backward stepwise selection of covariates having the lowest *P*-values, MUC1 > 40% and/or MUC4 > 0% expression independently predicted a poor prognosis (*P* = 0.043; hazard ratio 2.02, 95% CI 1.02, 3.98), adjusting for nodal involvement, vessel involvement and tumour size (Table 4).

**Table 3:** Unadjusted Cox regression analysis in pancreatobiliary differentiated adenocarcinomas

Positive tumour cells (%)	*N*	Hazard ratio	95% CI
CK20			
0 (ref)	27	1.00	–

0–10	22	1.09	0.59, 2.03

10–40	7	1.43	0.58, 3.55

>40	7	0.93	0.40, 2.18
MUC1			
0 (ref)	6	1.00	–

0–10	27	1.45	0.50, 4.22

10–40	12	2.13	0.67, 6.71

>40	18	2.82	0.94, 8.49

MUC4			
0 (ref)	21	1.00	–

0–10	23	1.96	1.01, 3.81

10–40	17	1.80	0.87, 3.75

>40	4	N/A	–

Hazard ratio >1 indicates increased risk of death compared with ref.

Ref, reference group; N/A, not applicable (due to small numbers); CK, cytokeratin.

**Table 4:** Adjusted Cox regression analysis in pancreatobiliary differentiated adenocarcinomas

		Hazard ratio	95% CI	*P*-value
MUC1 > 40% and/or MUC4 > 0%	Yes (versus no)	2.02	1.02, 3.98	0.043

Lymph node involvement	Yes (versus no)	3.53	1.72, 7.21	<0.001

Vessel involvement	Yes (versus no)	1.93	1.04, 3.59	0.037

Tumour size	>25 mm (versus ≤25 mm)	2.54	1.39, 4.63	0.002

In intestinally differentiated pancreatic head adenocarcinomas (*n* = 47), none of the differentiation markers CK7 (*P* = 0.62), CK20 (*P* = 0.85), MUC1 (*P* = 0.90), MUC2 (*P* = 0.43), MUC4 (*P* = 0.94) or CDX2 (*P* = 0.86) was significantly associated with survival.

## Discussion

Determination of the histological type based on the predominant pattern of histological differentiation^5^ may be performed with almost perfect interobserver agreement and could be a useful adjunct to classification of pancreatic head adenocarcinomas based on standard histopathological examination.^10^ The present study has demonstrated that classification of pancreatic head adenocarcinomas using common immunohistochemical markers associated with pancreatobiliary versus intestinal histological type is only in moderate agreement with classification based on morphological criteria. These markers may, however, provide prognostic information beyond indicating the histological type. Among patients with morphologically determined pancreatobiliary adenocarcinomas, immunohistochemical detection of MUC1 and MUC4 identifies a subgroup of patients with a particularly poor prognosis.

The three anatomical structures that may give rise to pancreatobiliary-type adenocarcinomas in the pancreatic head, i.e. the pancreas, ampulla and distal bile duct, are embryologically derived from the endodermal lining of the duodenum, as are periampullary tumours of intestinal differentiation. As they have a common tissue origin, it is not surprising that pancreatobiliary-type and intestinal-type adenocarcinomas may have overlapping expression of cellular markers, although differential expression of these markers in pancreatobiliary and intestinal-type tumours has frequently been reported.^6^,^8^,^17^,^18^,^22^–^25^,^39^ Determination of the histological type of differentiation in pancreatic head adenocarcinomas is important for a number of reasons. We have previously suggested that the histological type might provide more precise information regarding long-term survival than the anatomical site of tumour origin.^10^ Failure to exclude prognostically favourable types of adenocarcinomas, e.g. intestinal ampullary adenocarcinomas, may obscure survival predictions in studies of resected pancreatic head adenocarcinomas.^2^,^40^ A potentially important clinical implication of this is that selection of patients for chemotherapy and study protocols might be based on the tumour’s type of differentiation as well as tumour origin. As we^10^ and others^6^–^9^ have demonstrated, even ductal adenocarcinomas arising from the pancreas or distal bile duct may have intestinal histological type of differentiation.

To our knowledge, this is the first report of differentiation marker expression in a collective series of pancreatic head adenocarcinomas including all four subtypes with respect to anatomical origin. Previous investigators, although often not including all four subtypes in the analysis, have in general found that expression of CK7, MUC1 and MUC4 predominantly identifies tumours with pancreatobiliary differentiation and a poor prognosis, whereas expression of CK20, MUC2 and CDX2 predominantly identifies tumours with intestinal differentiation and a good prognosis.^6^,^8^,^17^,^18^,^21^–^25^,^39^ No particular combination of biomarkers has been established for the immunohistochemical classification of histological type. In the present study, we obtained at best moderate to substantial agreement with morphological classification even when using the marker combination optimally corresponding to the histological type. We defined the cut-off values for immunohistochemical classification prior to statistical analysis,^41^,^42^ as required if such markers are to be used in clinical practice or clinical studies. In previous studies, cut-off values discriminating between negative and positive samples have varied considerably, in the range 0–5%^6^,^22^,^26^,^32^,^43^ to 20–25%.^19^,^21^,^25^ Alternative cut-off values did not discriminate more precisely between pancreatobiliary and intestinal differentiation in the present study.^44^ However, further markers associated with the histological type of differentiation should be evaluated, and several cut-off values discriminating between poor and good prognostic groups should be explored for these markers. Other antibodies directed against the mucins MUC1, MUC2 and MUC4 should also be evaluated, since these proteins are heavily glycosylated, and altered glycosylation in cancer may influence the reactivity to such antibodies.^45^,^46^

Tissue microarrays could introduce selection bias of the histological type, particularly since differentiation markers are not always homogeneously expressed within a single tumour. However, only 10 tumours with both types of differentiation were identified in the study group consisting of 114 consecutive resections, and for each of these tumours with mixed-type histology, either pancreatobiliary or intestinal differentiation was readily identified as the predominant pattern. This was reflected in the high interobserver agreement in morphological classification of the histological type.

In the present study, MUC1 and MUC4 expression identified a subgroup of patients that had a particularly poor prognosis among patients with a differentiated pancreatobility tumour. Both MUC1 and MUC4 are membrane-associated mucins involved in cellular contact and signalling and may play a role in the autonomous and dysregulated proliferation seen in cancer cells.^47^ These mucin proteins are not necessarily expressed simultaneously,^21^ and the combined evaluation of MUC1 and MUC4 expression might therefore increase sensitivity in detection of prognostically poor tumours among these patients. The subgroup analysis examining the prognostic impact of MUC1 and MUC4 expression included 65 patients. Although this number is relatively small, most of these patients (*n* = 55) were dead by the end of the study, and only two of the eight censored patients in this subgroup were followed for <5 years (these were followed for 4.2 and 4.8 years, respectively). Actual 5-year survival was thus almost complete, and the high number of events during the study period made the subgroup analysis feasible. The results of this analysis were confirmed by Cox regression analysis adjusting for possible confounders, demonstrating that MUC1 and/or MUC4 expression was indeed an independent prognostic factor among patients with pancreatobiliary differentiated adenocarcinomas.

In conclusion, morphological classification of histological type significantly discriminates between prognostically poor pancreatobiliary and prognostically good intestinal types of pancreatic head adenocarcinomas. Agreement between immunohistochemical and morphological classification of pancreatic head adenocarcinomas is only moderate, and immunohistochemical characterization is thus not appropriate to discriminate between these two histological types. However, MUC1 and MUC4 expression may identify patients with a particularly poor prognosis among morphologically determined pancreatobiliary-type pancreatic head adenocarcinomas.

## Abbreviations

CI: confidence interval
CK: cytokeratin

## Supporting information

**Figure S1.** Bar graph demonstrating that the phenotypes CK7^+^ MUC4^+^ (CDX2^+^/^−^) and CK7^+^ CDX2^−^ (MUC4^+^/^−^) were predominantly pancreatobiliary (‘IHC pancreatobiliary’), while the remaining phenotypes, CK7^+^ (MUC4^+^/^−^) CDX2^+^/^−^) and MUC4^−^ CDX2^+^ (CK7^+^/^−^) were predominantly intestinal (‘IHC intestinal’).
